# Strain Monitoring of Vertical Axis Wind Turbine Tower Using Fiber Bragg Gratings

**DOI:** 10.3390/s25133921

**Published:** 2025-06-24

**Authors:** Bastien Van Esbeen, Valentin Manto, Damien Kinet, Corentin Guyot, Christophe Caucheteur

**Affiliations:** 1 Advanced Photonic Sensors Unit, University of Mons, 31 Boulevard Dolez, 7000 Mons, Belgium; christophe.caucheteur@umons.ac.be; 2 Fairwind s.a., 299 Chaussée de Gilly, 6220 Fleurus, Belgium; valentin.manto@fairwind.be; 3 B-SENS SRL, 31 Boulevard Dolez, 7000 Mons, Belgium; damien.kinet@b-sens.be (D.K.); corentin.guyot@b-sens.be (C.G.)

**Keywords:** fiber Bragg gratings, fiber optic sensor, VAWT monitoring, tower bending, structural health monitoring

## Abstract

This article presents the findings of an experimental study conducted on a vertical axis wind turbine (VAWT) tower instrumented with cascaded fiber Bragg grating (FBG) sensors to detect bending deformations. Structural health monitoring (SHM) is an essential need in the industry to reduce costs and maintenance time, and to prevent machine failures. First, FBG strain sensors were glued vertically along the tower to investigate the sensors behavior as a function of their height. The maximum signal-to-noise ratio is obtained when FBGs are placed at the tower base. Then, four packages were installed inside the tower, at the base, according to four cardinal directions. Each package contains an FBG strain sensor, and an extra temperature FBG for discrimination. The use of easy-to-deploy packages is a must for industrial installations. Afterwards, by using power spectral density (PSD) on the strain signals, three sources of tower oscillations are discovered: wind force, structure unbalance, and 1st tower mode resonance, each with its intrinsic frequency. Wind force and structure unbalance cause mechanical stresses at a frequency proportional to the wind turbine rotational speed, while the 1st tower mode frequency depends only on the machine geometry, regardless of the rotational speed. This study also analyzes the deformation amplitude for different rotational rates within the VAWT operational range (10–35 rpm). The resonance amplitude depends on the proximity of the rotational rate to the resonant frequency (22 rpm) and the duration at that rate. For structure unbalance, the oscillation amplitude increases with the rotational rate, due to the centrifugal effect. It is supposed that wind force deformation amplitude naturally depends on wind speed, which is unpredictable at a given precise time. The results of our experimental observations are very valuable for both the wind turbine manufacturer and owner.

## 1. Introduction

Structural health monitoring (SHM) of wind turbines is crucial to ensure their safe operation, optimize maintenance strategies, and reduce downtime costs [1,2]. In particular, their continuous monitoring allows the early detection of mechanical degradations such as blade imbalance, foundation defects, or structural resonance shifts. While traditional approaches often rely on accelerometers [3] or conventional strain sensors [4,5,6,7], fiber Bragg grating (FBG) sensors have emerged as a promising alternative due to their high sensitivity, immunity to electromagnetic interference, multiplexing capability, and long-term stability [8,9].

FBG-based SHM has been explored quite extensively for horizontal axis wind turbines (both onshore and offshore [10,11,12]), where sensors have been installed on blades [13,14,15,16], towers, and foundations (comprising monopiles for offshore wind turbines) to measure strain, temperature, and vibrations [17,18,19,20,21]. However, their application to vertical axis wind turbines (VAWTs), which present distinct dynamic behaviors and structural configurations, remains less investigated. An overview of the positioning of this paper can be found in Table 1, compared with other works.

In general, VAWTs, which are smaller than HAWTs, are produced for particular companies, not for large public energy production like HAWTs. The consequence is that VAWTs can be located in urban environments, where some obstacles are present in their proximity, leading to more turbulent and unpredictable wind, hence the necessity of monitoring the strain on such wind turbines. This can be seen as a drawback, but the shape of VAWTs offers the advantage of being independent on the wind direction, unlike HAWTs. Moreover, due to the geometry of a VAWT and the absence of electrical power on its machine head, the latter is impossible to monitor with a full-wired solution, which presents an extra challenge. These are the reasons why the specific use of FBG strain gauges located at the base of the tower, for indirect monitoring of turbine health parameters, offers a valuable, cost-effective, and easy-to-deploy alternative to conventional vibration-based methods.

In this work, we report on the instrumentation of a VAWT using FBG strain gauges to assess the structural dynamics of the machine. Initially, bare optical fibers with FBGs were directly glued on the external side of the tower at various heights. Our results show that the sensor closest to the tower base exhibits the highest signal-to-noise ratio (SNR), confirming the relevance of locating sensors near maximum strain regions.

To further improve measurement reliability and allow strain-temperature discrimination, we developed custom 3D-printed sensor packaging. Each package embeds two wavelength-division-multiplexed FBGs: one strain-sensitive and one isolated from strain to act as a temperature reference. These sensor pads were then installed inside the tower base in four cardinal directions (north, south, west, east). The time series analysis of the FBG signals revealed that mirror sensors (e.g., north vs. south) exhibit antagonist strain behaviors, as expected from tower bending under asymmetric loads.

Through spectral analysis (power spectral density—PSD) of the recorded strain signals, we found out several key frequencies that give useful information on the wind turbine state and conditions. These frequencies correspond to the real-time behavior of the machine, some of them depend on the rotational speed, while another one reflects a natural resonance mode of the structure.

Our findings demonstrate that FBG strain gauges positioned at the bottom of the tower can effectively replace traditional top-mounted accelerometers, offering a compact, cost-effective, easy-to-install, and highly sensitive solution for SHM of VAWTs.

## 2. Materials and Methods

### 2.1. Studied Machine

**Figure 1:**
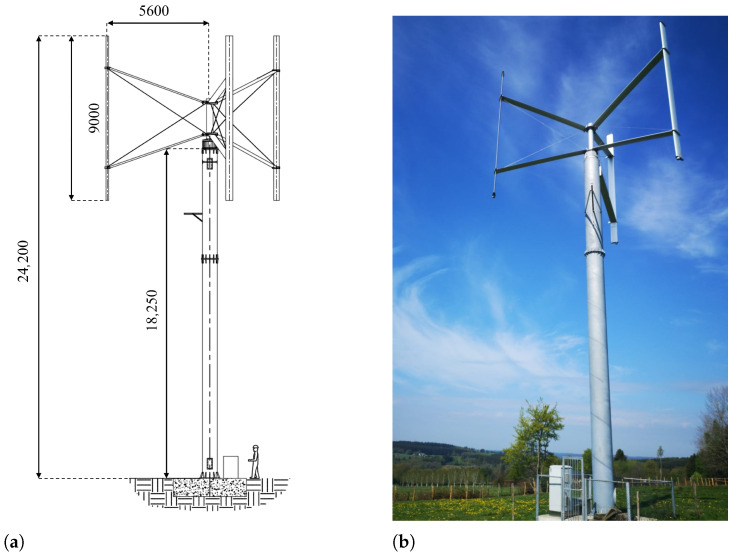
F100-10 VAWT: (**a**) scheme with measurements in mm, (**b**) picture.

The studied vertical axis wind turbine is the model F100-10 of Fairwind (Fleurus, Belgium). It is a machine of 24.2 m in height, characterized by a power of 10 kW. A scheme of this VAWT can be found in Figure 1a, where the measurements are expressed in mm, and a picture of the machine in Figure 1b.

### 2.2. Sensors

An FBG is a permanent modification of the refractive index in the core of an optical fiber, along its propagation axis. This modification follows a periodic pattern, leading to a grating of length $L_{G}$ and period Λ. It acts as a distributed mirror that reflects a part of the incident light around the Bragg wavelength $\lambda_{B}$, which is obtained by:

(1) $$\lambda_{B}=2n_{eff}\Lambda$$

with $n_{eff}$ as the effective refractive index of the core. The grating period has to be about 530 nm to obtain a Bragg wavelength around 1550 nm. The FBG sensing principle relies on the fact that any change of temperature or axial strain will induce a variation of $n_{eff}$ and Λ, inducing a shift of the Bragg wavelength $\lambda_{B}$. The sensitivity of the Bragg wavelength shift $\Delta \lambda_{B}$ behaves linearly and without hysteresis; typical values at a wavelength of 1550 nm are 1.2 pm/µε and 10 pm/°C for axial strain and temperature sensing, respectively, [9]. When both strain and temperature changes happen simultaneously, a discrimination is necessary to determine the contribution of the physical phenomenon that needs to be measured.

An important advantage of this technology is the possibility to cascade several gratings at different Bragg wavelengths in a single optical fiber. Every Bragg wavelength shift can be measured simultaneously provided that the optical source and the data acquisition system (also called interrogator) are suitable for wavelength division multiplexing.

The gratings used in this work were manufactured using the NORIA facility (Northlab Photonics, Nacka, Sweden). This device contains a 193 nm excimer laser and uses the phase mask technique [22] to produce FBGs with a 50 Hz repetition rate of laser pulses. FBGs of 6 mm in length were inscribed into single-mode photosensitive optical fibers from the company Fibercore (Southampton, UK) (fiber type PS1250/1500). The Bragg wavelengths are distributed between 1510–1580 nm.

### 2.3. Installation

This study focuses on tower bending deformations. In this case, the strain at the tower surface happens vertically and, as FBGs are sensitive to axial strain, the optical fiber has to be glued vertically on the tower. As explained above, these sensors are also affected by temperature changes. A discrimination can be performed by adding another FBG, at a different Bragg wavelength, only sensitive to temperature (free to move). The Bragg wavelength shift of the latter can be subtracted from that of the strain sensors to infer the axial strain contribution.

Two different tests were conducted: one with the sensors placed outside (that will be called outside test in the remainder of the document), and one with the sensors placed inside the tower (called inside test). These are detailed in the next sections.

#### 2.3.1. Outside Test

**Figure 2:**
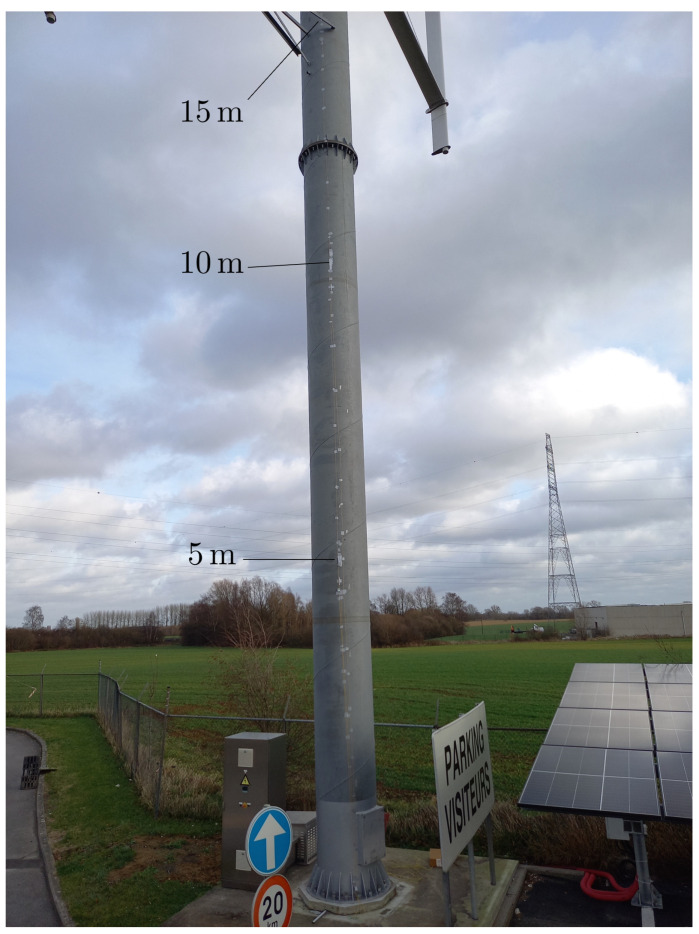
Picture of the FBGs installed on the VAWT tower surface.

Four sensors were made for the outside test. Among them, three were glued, bare, on the tower surface with a view to strain sensing, while a protective sheath was placed around the fourth grating, acting as temperature sensor. According to the principles of materials resistance theory, in the scenario of simple plane bending (let us consider the case of a vertical beam embedded at the base and free at the top, i.e., a vertical cantilever beam), the elongation of longitudinal elements at the beam surface can be easily found:

(2) $$\varepsilon =\frac{P\left(L-x\right)a}{2EI_{z}}$$

where *P* is the applied load at the free end of the beam, *L* its length, *x* the height at which the elongation is computed, *a* the beam width, *E* its Young’s modulus, and $I_{z}$ the moment of inertia. As *x* can take values between 0 and *L*, the maximum elongation occurs at x=0, i.e., at the base of the beam, or the VAWT tower in our case. However, this is based on different hypotheses: the only deformation taken into account is simple plane bending, there is only one load applied at the very top of the tower, the tower is the only element considered in the model (blades are neglected), and the VAWT tower is perfectly embedded in the ground. In practice, real conditions might not reflect the theoretical models very well. Therefore, to assess the validity of our hypotheses, the three strain FBGs were installed at different heights (5, 10 and 15 m) with cyanoacrilate adhesive, as shown in Figure 2. These heights are not accessible inside the tower, so the sensors were placed outside. The side corresponding to the prevailing wind direction was chosen. Before gluing, a small area on the tower surface was sanded and degreased to allow a good and clean contact between each FBG and the tower. These sensors were installed in a pre-strained way to ensure compression sensitivity, and a fourth grating (not glued) was foreseen for the sake of temperature sensing.

#### 2.3.2. Inside Test

**Figure 3:**
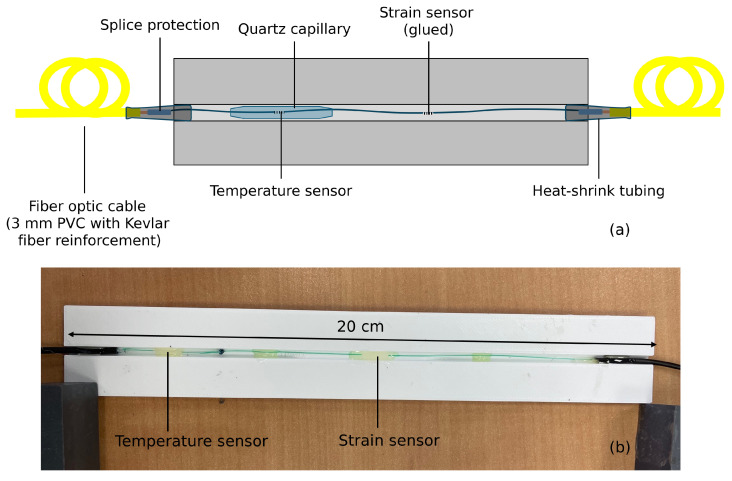
Package for the inside installation, with one strain and one temperature FBG: (**a**) scheme, (**b**) picture.

Regarding the inside test, four packages were built, and one of them is shown in Figure 3. The supports were 3D-printed in PLA. Each support has a groove in which a fiber had been placed. The fiber is composed of two FBGs: one for strain measurement (glued to the support inside the groove) and one for temperature compensation (left free in a Quartz capillary). The package is 20 cm long so that it makes it an easy-to-handle package, and there were no particular space requirements. The packages were installed inside the tower, at its bottom (at ∼2 m) to maximize the elongation and thus the Bragg wavelength shift (see Section 3.1), and at 90° from each other (north, south, west, east). The packages ensure easier installation because the FBGs could be pre-strained in the lab instead of directly on the tower, and the gluing was performed on an easy-to-handle surface instead of the bare fiber. Moreover, placing the sensors in a package and in an indoor environment eliminates any risk of alteration due to humidity [23].

## 3. Results and Discussion

Measurements consist of recording the Bragg wavelength shift of the sensors as a function of time. This is achieved using an FBG interrogator at a sampling frequency of 100 Hz and a spectral resolution of 1 pm (after post-processing). This interrogator, called BSI-108 from the company B-SENS (Mons, Belgium), operates using a broadband optical source and a spectrometer.

### 3.1. Outside Test

**Figure 4:**
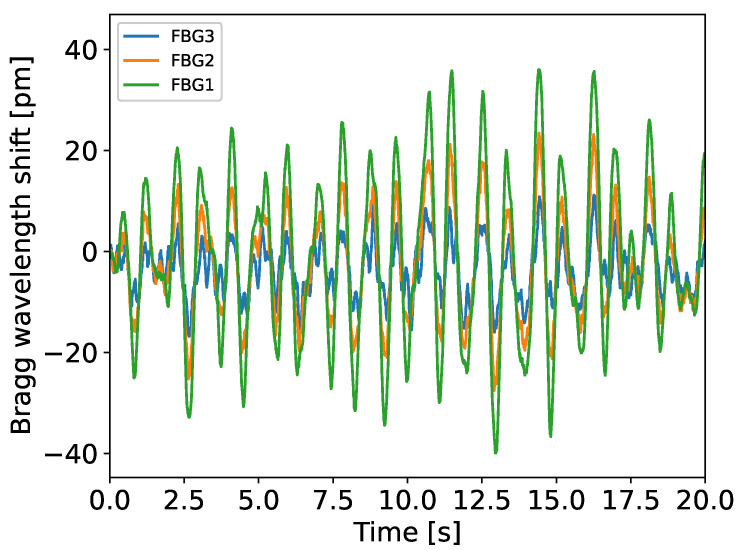
Temporal evolution of the Bragg wavelength shifts for FBG 1, 2, and 3 placed at 5, 10, and 15 m, respectively.

An evolution of the Bragg wavelength shift of the three strain sensors, placed at 5, 10, and 15 m, when the VAWT rotated at around 33 rpm (rotations per minute), can be found in Figure 4, for a duration of 20 s. The temperature during this measurement was around 15 °C.

**Figure 5:**
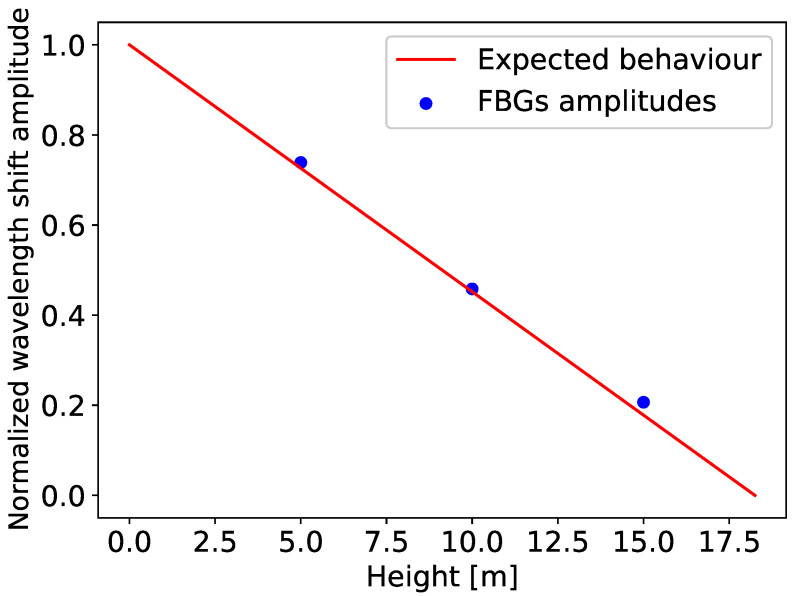
Comparison between the expected behavior (red) and the normalized Bragg wavelength shift amplitude (blue) for the three FBGs (placed at 5, 10, and 15 m), as a function of the height along the tower.

According to the theoretical model, the amplitude of the signals should follow a straight line with respect to the height, with the maximum amplitude at the bottom. Figure 5 represents the normalized amplitude of the FBGs’ wavelength shift (blue dots). The straight line (red) characterizes the expected behavior based on theory (with *L* = 18.25 m, see Figure 1a). This observation confirms that reality reflects the model very well, even with the hypotheses that were made. In conclusion, in the case of bending monitoring, FBGs should be placed at the bottom of the VAWT tower to maximize their signal-to-noise ratio.

### 3.2. Inside Test

**Figure 6:**
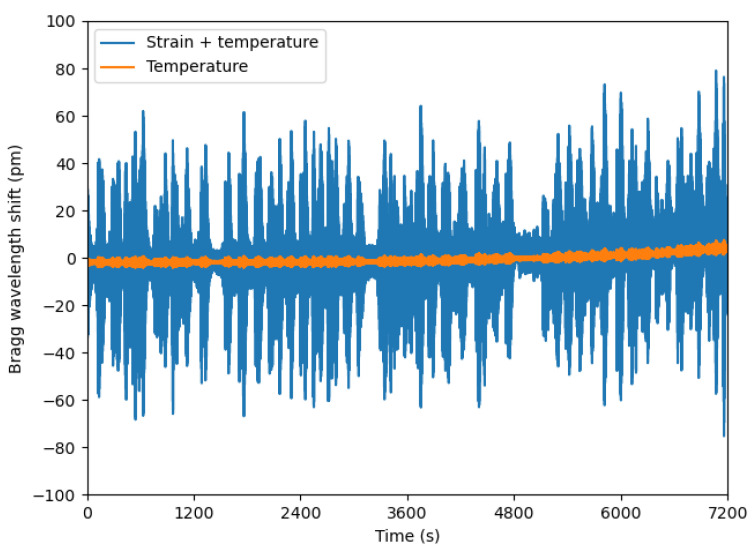
Evolution of the Bragg wavelength shifts of two FBGs embedded in one package vs. time. Orange represents the temperature change and blue the strain and temperature changes.

The signal recorded by both FBGs of one package can be found in Figure 6, for a duration of two hours (7200 s).

**Figure 7:**
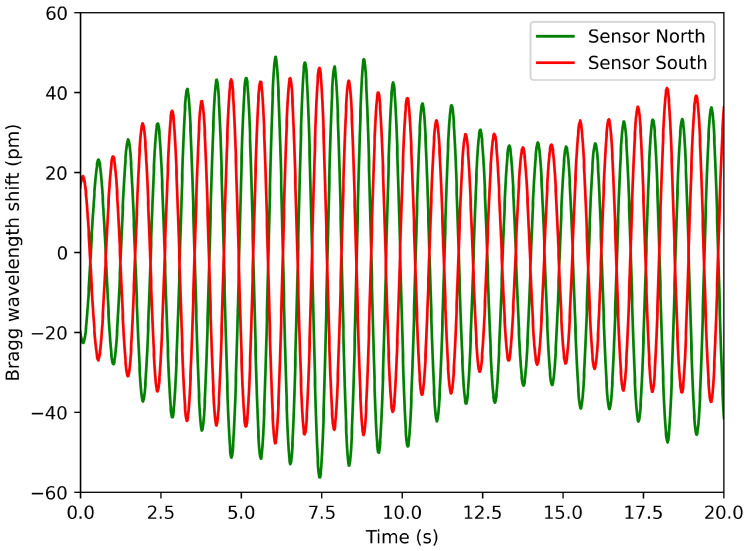
Strain signal vs. time of two sensors installed at opposite sides.

The useful information (strain) is inferred by subtracting the orange signal from the blue signal. Figure 7 represents the strain evolution for a duration of 20 s, for two opposite sensors (north and south).

As expected, both signals are perfectly in phase opposition. The same observation can be made for the west–east sensors. By comparing every signal with each other, we have a lot of information on the orientation of the bending. Furthermore, defects on a specific side of the tower could be quickly detected if the amplitude of one strain signal became larger than its opposite.

### 3.3. Frequency Analysis

#### 3.3.1. Power Spectral Density

In this section, a frequency analysis is performed on the strain signals obtained from one sensor. The analysis is carried out by imposing different rotational speeds on the VAWT generator. The operating range of the F100-10 wind turbine lies between 10 and 35 rpm. According to the Fairwind technical report, there is a resonance phenomenon when the machine vibrates at 1.1 Hz, coinciding with a rotational speed of 22 rpm. With that in mind, measurements are taken to explore a large set of mechanical behaviors within the entire operating range. The imposed speeds range from 10 to 35 rpm with steps of 5 rpm. Right before the resonance frequency, steps of 1 rpm are implemented between 15 and 20 rpm to evaluate the behavior evolution more precisely. Naturally, for safety reasons, the passage between 20 and 25 rpm must be as quick as possible to avoid too much deformation that could cause mechanical damage.

**Figure 8:**
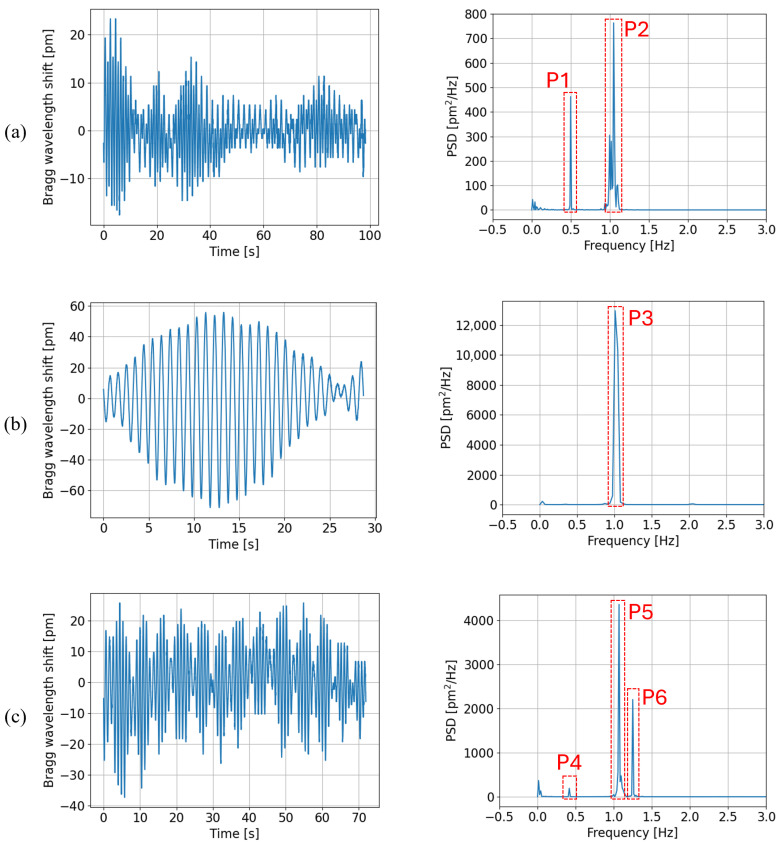
(**Left**) Bragg wavelength shift induced by strain vs. time at rotational speeds of (**a**) 10 rpm, (**b**) 20 rpm, (**c**) 25 rpm. (**Right**) Corresponding power spectral density computations.

The frequency analysis consists of computing the power spectral density (PSD) of the strain signal for every imposed rotational speed. The result at three speeds (10, 20, and 25 rpm) can be found in Figure 8, with the strain signals on the left and the corresponding PSD computation on the right.

Based on the peaks’ positions and amplitudes, three distinct phenomena were inferred from the PSD graphs. The analysis was conducted on all the results even though this paper only shows the graphs for three rotational rates. First, the tower bends under the wind force. The magnitude of the bending deformation due to wind force depends on the wind speed and on the position of the blades compared to the wind direction. As the VAWT machine head is composed of three blades, there are three repetitions of the blades’ position per rotation. Therefore, the wind force does not create a constant tower bending but oscillations at a frequency of $\left(R/60\right)\;\cdot \;3$ Hz with *R* the VAWT rotational rate in rpm.

Secondly, in the theory of vibrating machines, attention must be paid to the resonance phenomena. As mentioned above, the machine shows a resonance mode at about 1.1 Hz called the 1st tower mode and characterized by a bending mechanical deformation. Hence, at a rotational rate of 22 rpm, the wind force would amplify a repeated mechanical force at $\left(22/60\right)\;\cdot \;\;3=$ 1.1 Hz, exciting the 1st tower mode and thus creating tower bends of increasing amplitude at a frequency of 1.1 Hz. As a consequence, since 22 rpm lies in the VAWT operating range (10–35 rpm), any passage through this rotational rate must be quick to avoid amplification of the 1st tower mode response that could cause serious damage. The amplitude of oscillations at 1.1 Hz depends thus on the elapsed time: there is amplification if the rate is 22 rpm, fading if not.

Thirdly, the last detected source of tower bending is the structure unbalance. In an ideal world, wind turbines are perfectly balanced but some weight differences are unavoidable in practice. This unbalance effect is more and more important as the rotational speed increases, due to the centrifugal effect. The bending created by the unbalance happens at the structure rotational rate, i.e., R/60 in Hz.

A summary of these bending sources can be found in Table 2, where the frequency analysis is quantitatively oriented and the amplitude qualitatively. These observations are relevant for any VAWT model, as long as the resonance frequency is adapted considering the studied machine.

These three physical phenomena explained above, identified as the tower bending sources, can be observed on the PSD graphs. In Figure 8a right (10 rpm), a first peak (P1) is located at 0.5 Hz, attesting the presence of oscillations due to wind force because $\left(10/60\right)\;\cdot \;3=0.5$. The second peak (P2) at 1.1 Hz reveals a passage through 22 rpm shortly before this measurement session. In Figure 8b right (20 rpm), both peaks at $\left(20/60\right)\;\cdot \;\;3=$ 1 Hz and at 1.1 Hz are so close to each other that they overlap (P3). Finally, Figure 8c right (25 rpm) shows peaks at $\left(25/60\right)\;\cdot \;\;3=$ 1.25 Hz (P6), at 1.1 Hz (P5), and at ∼ 0.42 Hz (P4). The latter stands for the oscillations due to the structure unbalance, meaning that 25 rpm becomes non-negligible for the centrifugal effect, indeed 25/60≈ 0.42 Hz.

#### 3.3.2. Spectrogram

**Figure 9:**
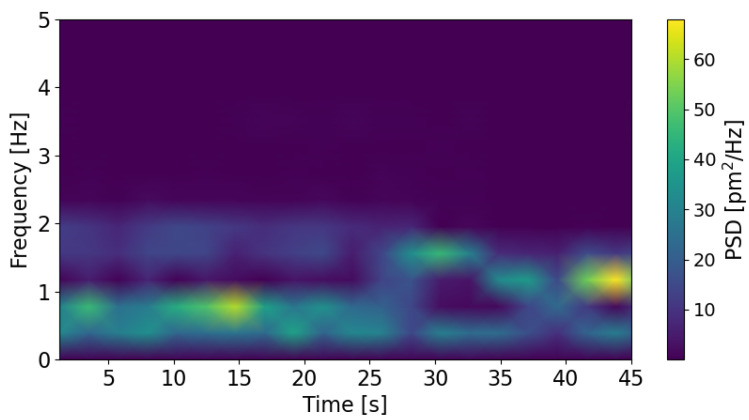
Spectrogram measured when the VAWT rotates from 35 down to 27 rpm.

The limitation of power spectral density is the assumption that the signal is periodic and infinitely long. In practice, the frequencies of the tower oscillations change with time. Therefore, computing the spectrogram of the signal is a good alternative to show the evolution of the frequencies as a function of time. Figure 9 shows the spectrogram of the measured signal when the rotational rate of the VAWT generator was imposed from 35 down to 27 rpm by steps of 1 rpm every 5 s.

In this example, the evolution is clear. First, at high rotational rate, from 35 to 31 rpm (from 0 to 25 s), the main source of oscillation is the structure unbalance (R/60 Hz), and the oscillations due to wind force are also visible (R·3/60 Hz). Then, from 30 to 28 rpm (from 25 to 40 s), the centrifugal effect fades away, and the main contribution becomes the oscillations due to wind force. At the end, the rotational rates approach more and more the resonance frequency (1.1 Hz).

## 4. Conclusions

In this work, it has been demonstrated that fiber Bragg gratings sensors are a valuable technology for VAWT strain monitoring. Sensors design and installation were performed on a VAWT in Fleurus, Belgium. Two tests were conducted: the outside test with bare strain FBGs installed at 5, 10, and 15 m on the tower surface, and the inside test with four packages of two FBGs (strain and temperature) placed at 90° from each other. The first confirmed that strain sensors should be placed at the base of the tower to maximize the SNR, and the second demonstrated the viability of the FBG technology, and the benefits of easy-to-install packages. Then, a frequency analysis was carried out at different rotational speeds imposed on the VAWT generator, within its operational range (10–35 rpm). Three sources of tower bending were found out: the wind force, the 1st tower mode response, and the structure unbalance. Each of these phenomena is characterized by a specific frequency. First, the wind force implies bending oscillations at R[rpm]/60·3 Hz, due to the fact that the blades position repeats thrice per rotation. Then, the resonance frequency (due to the 1st tower mode) only depends on the machine geometry, so it is constant for a given VAWT (here, it is 1.1 Hz). Finally, the structure unbalance induces tower bends at the rotational speed, i.e., R[rpm]/60 Hz. 

## Figures and Tables

**Table 1:** Overview of the strain monitoring approaches for both HAWTs and VAWTs.

	Traditional Approaches	FBG Technology
HAWT	[3,4]	[10,11,12,13,14,15,16,17,18,19,20,21]
VAWT	[5,6,7]	This work

**Table 2:** Overview of the bending deformation sources in terms of frequency and amplitude.

	Wind Force	1st Tower Mode	Structure Unbalance
Frequency [Hz]	(R[rpm]/60)·3	1.1	R[rpm]/60
Amplitude (trend)	Depends on wind speed	Depends on *R* (close to 22 rpm or not) and on elapsed time	Proportional to $R^{2}$ due to centrifugal effect

## Data Availability

The raw data supporting the conclusions of this article will be made available by the authors on request.
